# Microbial cohorts: bringing ecological meaning to the modularity concept of co-occurrence networks

**DOI:** 10.1093/ismeco/ycag037

**Published:** 2026-02-21

**Authors:** Felix Milke, Sarahi L Garcia, Meinhard Simon, Armando Pacheco-Valenciana, Sinikka T Lennartz

**Affiliations:** Institute for Chemistry and Biology of the Marine Environment (ICBM), School of Mathematics and Science, Carl von Ossietzky University Oldenburg, 26129 Oldenburg, Germany; Institute for Chemistry and Biology of the Marine Environment (ICBM), School of Mathematics and Science, Carl von Ossietzky University Oldenburg, 26129 Oldenburg, Germany; Department of Ecology, Environment, and Plant Sciences, Science for Life Laboratory, Stockholm University, SE-106 91 Stockholm, Sweden; Helmholtz Institute for Functional Marine Biodiversity at the University of Oldenburg (HIFMB), 26129 Oldenburg, Germany; Institute for Chemistry and Biology of the Marine Environment (ICBM), School of Mathematics and Science, Carl von Ossietzky University Oldenburg, 26129 Oldenburg, Germany; Helmholtz Institute for Functional Marine Biodiversity at the University of Oldenburg (HIFMB), 26129 Oldenburg, Germany; Department of Ecology, Environment, and Plant Sciences, Science for Life Laboratory, Stockholm University, SE-106 91 Stockholm, Sweden; Institute for Chemistry and Biology of the Marine Environment (ICBM), School of Mathematics and Science, Carl von Ossietzky University Oldenburg, 26129 Oldenburg, Germany

**Keywords:** microbial ecology, co-occurrence networks, biological organization, modularity, microbial community assembly, ecological niche, microbiome, biodiversity, meta-analysis, biogeography

## Abstract

Microbial communities are structured through complex interactions that are difficult to observe directly. Co-occurrence networks offer a way to infer community structure, revealing (not exclusively) potential biotic interactions. Such networks have been inferred for diverse biomes and repeatedly found to be modular, yet the ecological significance of this modularity remains underexplored. We tested whether clusters within co-occurrence networks (“cohorts”), are universal and ecologically meaningful units by assessing their ubiquity, stability, and environmental specificity across diverse ecosystems. Our meta-analysis spans 25 previously published 16S rRNA gene amplicon sequencing datasets (14 160 samples) and covers high environmental variability ranging from aquatic, terrestrial to anthropogenic environments. Microbial co-occurrence networks consistently exhibited high modularity across biomes. Inferred cohorts were ubiquitous and represented up to 90% of the community composition. Our findings demonstrate that modularity is a fundamental and generalizable feature of microbial community organization, indicating the existence of stable subcommunities. Highly similar cohorts were inferred even across different, unconnected environments and datasets, and showed consistent responses to environmental gradients, indicating that their composition is to a large degree deterministic and predictable. The overall cohort structure and environmental preferences were independent of the sample size and the inference algorithm, underlining the robustness and applicability of the results. Recognizing these microbial cohorts as a meaningful level of microbial organization will refine microbial community ecology, cultivation strategies, and predictive modelling of microbial dynamics.

## Introduction

Microorganisms are the basis of global food webs, drive essential nutrient cycles, and influence global climate regulation through their diverse metabolic capabilities [1]. The great majority of microbial communities in the environment is characterized by their enormous diversity [2]. Microorganisms form a tight interacting network in the environment that enables them to perform complex tasks such as degrading complex sugars [3, 4] or naturally occurring biopolymers [5, 6]. These interactions are ubiquitous and comprise mutualism, commensalism, but also competition, predation and antagonistic interactions [7]. However, unlike in the metazoan realm, these functional interactions are difficult to observe directly, hampering our understanding of the structure of microbial communities and the underlying functions and mechanisms of their assembly in natural systems. Since microbial interaction structure beyond compositional data is largely unobservable, a common approach is to determine community organization through co-occurrence patterns in microbial abundance data. Such inferred networks consistently exhibit modular structures, with these modules described by various names across studies: modules [8], metabolically cohesive consortia [9], co-occurring assemblages [10], functional cohorts [11], microbial active functional modules [12], interactive guilds [13], and community types [14]. Despite the terminological diversity, all describe the same core concept: microbial subcommunities whose members consistently co-occur and potentially function together. Such subcommunities have been detected across a wide range of environments, including mangrove sediments [12], high-mountain lacustrine plains [13], global oceans [8], lakes [11], and human body sites [14]. Yet, whether these statistically inferred clusters truly represent ecologically meaningful and generalizable cohorts remains an open question.

The ecological implications of generalizable modularity in microbial communities would be profound, highlighting the need for a cross-biome meta-analysis. Modularity suggests the existence of subcommunities: cohorts of microbial populations that consistently co-occur and share similar ecological niches. A generalizable modularity in microbial communities suggests a kind of “key-lock” exclusivity, where microbial populations preferentially assemble within particular cohorts that exhibit spatial and temporal stability. Supporting the key-lock principle, it was shown that metabolic exchange is more likely within than between co-occurring populations [15, 16].

If microbial communities consistently assemble into stable cohorts, this raises important questions for empirical and theoretical microbiology: Currently, experimental microbiology faces a severe culturing bias, focusing on the minuscule fraction of microorganisms that have been easily and axenically cultivated [17]. Instead of focusing solely on pure cultures, we need to embrace the full spectrum of microbial organization to better understand microbial capabilities and interactions as they occur in nature, especially considering that their cultivability increases if we cultivate them in groups [18]. Recognizing and studying these ecologically meaningful groupings is key to bridging laboratory observations with environmental reality. Scaling this understanding to ecosystem and global levels requires predictive models to aggregate microbial diversity into such meaningful groups. An understanding of community structure linked to functional traits could be a step-change for species distribution or Earth System Models.

En route to such an understanding, we determined whether microbial cohorts are ecologically meaningful entities, moving away from the purely statistical inference towards ecological relevance. We define cohorts from here on as cluster in co-occurrence network topologies. We assessed three criteria to determine their ecological relevance, i.e. (i) their ubiquity in nature, with the majority of microbial individuals being assignable to a cohort, (ii) their compositional stability even across unconnected locations and environments, and (iii) their distinct ecological preferences. To do so, we assembled a comprehensive collection of 25 microbial community datasets from aquatic, terrestrial, animal and anthropogenic habitats, comprising 14 160 16S rRNA gene amplicon samples. We validated our methodological approach by testing different combinations of data subsets (soil: 3 random subsets; ocean: systematic Ocean basin subsets) and different co-occurrence inference methods (SparCC, SpiecEasi, and Pearson). Our analysis revealed that the inferred co-occurrence networks consistently exhibit significantly greater modularity than expected by chance, and that these modules are indeed stable across geographically unconnected locations or independent datasets. Finally, we found that cohort composition strongly correlates with environmental conditions, and that the extremes of environmental gradients tend to be dominated by fewer microbial cohorts, leading to a decline in modularity under more “stressful” conditions. We therefore propose microbial cohorts as an ecologically meaningful level of the hierarchical organisation of microbial communities.

## Materials and methods

### Collection and curation of amplicon datasets

To conduct robust co-occurrence network analyses, we compiled a diverse set of large-scale 16S rRNA gene amplicon sequencing datasets that describe prokaryotic communities within specific environments. Given that co-occurrence inference requires a high number of samples to ensure statistical reliability, we selected datasets that contained a sufficiently large (minimum 65 samples, average 566 samples per dataset) and coherent sample set from a given environment.

A primary resource for environmental microbiome data was the Earth Microbiome Project (EMP), which provides thousands of samples across various habitats [19]. To construct robust co-occurrence networks, we refined the dataset to include only subsets of specific biomes that had sufficient data coverage (see Supplementary methods S1). We further incorporated data from other large-scale sequencing projects of oceanic, soil and freshwater environments. Specifically, we selected datasets from the San Pedro Ocean Time Series (SPOT) [20], the Australian Microbiome Project [21], the Californian Cooperative Ocean Fisheries Investigations (CalCOFI) [22], comparable latitudinal ocean transects (short: comparison dataset) [8] and the Lake Mendota time series [23]. After network inference and clustering, we compared network clusters from different environments to see if the same clusters can be found in different environments, which we interpreted as compositional stability (see below).

### Construction of co-occurrence networks

To infer significant positive co-occurrences among microbial taxa, we employed the SparCC algorithm [24], implemented via the computationally efficient FastSpar adaptation [25].

Prior to network inference, each dataset was filtered to retain all ASVs present in at least 10% of samples, while ensuring a minimum of the 5000 most abundant 16S rRNA gene amplicon sequence variants (ASVs) based on their mean abundance across all samples. The proportion of reads retained after applying this filter was >90% for most of the datasets (except the Australian Soil dataset, which consisted of >100 000 ASVs due to its large spatial scale). The total number of ASVs and proportion of reads retained after filtering is reported in Supplementary Table S1. Co-occurrence inference was conducted using FastSpar with 20 iterations per dataset. To assess statistical significance, we performed 500 bootstrap iterations, generating null distributions of correlation values. *P*-values were computed as the proportion of bootstrap iterations that produced correlation coefficients equal to or greater than the observed values, following the recommendations of Friedman & Alm [24]. These analyses were conducted using the fastspar_bootstrap and fastspar_pvalues functions in FastSpar (version 1.0.0). The resulting correlation and *P*-value matrices were imported into R (version 4.3.2) for subsequent network analyses. Correlation values associated with *P* > .05 were excluded from further analysis to account for false discovery rate (FDR). The filtered correlation matrix was then used as an adjacency matrix to construct a weighted, undirected co-occurrence network using the igraph package (version 2.0.3), with correlation coefficients serving as edge weights. Networks were thresholded by retaining only edges with correlation values above a predefined r-threshold (most analyses used here r = 0.25 or r = 0.4), bracketing values from the original SparCC publication (r = 0.3) [24]. Nodes without edges were removed from subsequent analyses.

### Network sensitivity analysis to varying r-thresholds

To evaluate the sensitivity of co-occurrence network properties to varying correlation thresholds (r-thresholds), we constructed networks using r-thresholds ranging from 0.1 to 0.7 in increments of 0.01. Each network was characterized based on four key metrics: (i) clustering coefficient, (ii) modularity, (iii) average number of cluster members, and (iv) total number of inferred clusters. Additionally, we visualized the density distribution of r-values for each dataset within the same figure (Fig. S3). The clustering coefficient (i) was computed using the transitivity() function from the igraph package. Network clusters in this analysis were identified using the cluster_walktrap() function, from which we derived network modularity (ii), the average number of cluster members per cluster (iii), and the total number of inferred clusters (iv).

### Clustering of co-occurrence networks

Since we define microbial cohorts as groups of co-occurring organisms, clustering of co-occurrence networks is a critical step. Network clustering is commonly formulated as an optimization problem aimed at maximizing network modularity [26]. However, biological datasets typically exhibit high levels of noise, and microbial communities often contain overlapping clusters, complicating clustering outcomes [27]. The latter issue has been well documented in marine microbiomes, where cluster biogeography exhibited partial overlap in ecological transition zones [8], leading to ambiguous cluster boundaries. To address these challenges, we exploited the parametrization of the Leiden-clustering algorithm [28] to control clustering resolutions. Higher resolutions yield numerous smaller clusters, whereas lower resolutions produce fewer, larger clusters. To enhance differentiation among overlapping clusters, we implemented a consensus-based clustering approach that evaluates multiple resolutions and determines an optimal clustering solution. Such a consensus clustering approach has been previously shown to improve the stability and accuracy of network clustering [29]. A more detailed description of the clustering pipeline can be found in the supplementary methods (including Supplementary Fig. S1).

### Statistical significance of cluster membership

To assess the significance of inferred clusters, we evaluated whether the observed network modularity could be reproduced in randomly rewired networks. This was achieved by repeatedly computing network modularity across 500 iterations of rewired networks. Cluster identification was performed using a greedy modularity optimization algorithm [30]. We reasoned that the validation of inferred clusters should be based on a comparison of modularity scores between observed and randomized networks that maintains the same number of clusters. Hence, we set the number of clusters in the randomized networks to match that of the observed network. Rewiring was performed while preserving the original node degree distribution, using rewire iterations with 10 times the number of network-nodes. The statistical significance of the observed modularity was determined by calculating the proportion of modularity values from rewired networks that were equal to or greater than the observed modularity. This approach allowed us to assess whether the inferred network modularity could be achieved by random chance.

### Quantitative characterization of microbial cohorts

**Figure 1:**
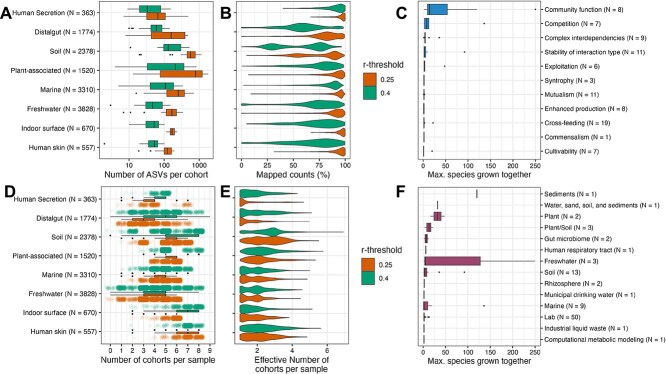
Ubiquity and properties of microbial cohorts and culture-bias against cohort sizes. (A, B, D, E) depict properties of microbial cohorts identified in 25 different datasets. Datasets were grouped into biome categories based on a modified EMP ontology and visualized as coherent groups with number of samples N shown in brackets. (A) boxplot showing the number of ASVs clustered into each cohort identified in the respective group of datasets. (B) violin plots showing the proportion of counts per sample that were mapped to a cohort. (D) boxplots with jittered dot-plots showing the number of microbial cohorts that were detected in a single sample. The number of cohorts is a discrete scale with round numbers and the jittered dots represent its density. (E) violin-plots showing the effective number of microbial cohorts per sample, which is the inverse Simpson-diversity of cohorts. This number takes into account the evenness of cohort abundance within a sample. (C, F) Boxplots showing the maximum number of strains/populations grown together in recent studies using culturing techniques. (C) shows the different ecological concepts that were tested in reviewed studies, whereas (F) shows the habitats of the isolates. Numbers N in brackets in c and f show the number of studies reviewed.

We quantitatively characterized microbial cohorts using four distinct metrics: (i) the number of ASVs per cohort, (ii) mapped counts, (iii) the number of cohorts per sample, and (iv) the effective number of cohorts per sample (Fig. 1A, B, D, E). These metrics were computed across all 25 datasets and for the two correlation thresholds (r = 0.25 and r = 0.4). By standardizing the number of ASVs per dataset, applying predefined correlation thresholds, and utilizing our advanced clustering pipeline, we improved comparability between datasets.

The number of ASVs per cohort (i) represents the number of inferred members within each network cluster. The proportion of mapped counts (ii) was calculated for each sample by determining the fraction of total sequence counts associated with ASVs belonging to cohorts. The number of microbial cohorts per sample (iii) corresponds to cohort richness and is expressed as discrete values, without accounting for the evenness of cohort composition within a sample. To address this limitation, we also computed the effective number of microbial cohorts per sample (iv), which incorporates cohort evenness. This metric was derived using the inverse Simpson diversity index, as implemented in the diversity() function of the R package vegan (version 2.6–4).

### Compositional stability of microbial cohorts

We defined compositional cohort stability as the degree of compositional similarity between independently inferred network clusters. To assess this, we manually selected datasets for comparison based on the presence of similar microbial taxa. Specifically, we compared network clusters from all distal gut datasets (human, monkey, deer, rabbit, and kangaroo), as they likely share similar ASVs. Additionally, we examined a second comparison group comprising datasets from two distinct studies: one containing microbiome samples from indoor surfaces, as well as skin and nasal secretions of inhabitants [31], and the other consisting of saliva samples from obese individuals [32]. These comparisons were chosen to demonstrate that similar or identical microbial cohorts can be inferred independently across (i) different hosts, (ii) different environments, and (iii) different studies.

Cohort composition was determined as the average ASV composition of a cohort within its respective dataset. Compositional similarity between microbial cohorts was quantified using Bray–Curtis similarity, calculated via the vegdist() function from the R package vegan. Pairwise similarities between all cohorts from the selected datasets were computed and visualized as a heatmap using the pheatmap package (version 1.0.12).

### Resilience of network clusters to perturbation

We assessed the resilience of inferred network clusters to perturbation by simulating targeted attacks through node removal (see Fig. S2 for a depiction of the analysis framework). Node removal was chosen as the attack mode because it mimics local extinctions of microbial populations, making it a more biologically meaningful perturbation compared to edge removal. To evaluate resilience, we systematically increased the proportion of removed nodes from 0% to 80% in 5% increments. Each perturbation scenario was bootstrapped 50 times, with network nodes randomly selected for removal in each iteration. The resulting networks were subsequently clustered using the Leiden algorithm [28]. Since the network attack was bootstrapped to account for random node selection, we did not reapply the entire clustering pipeline for each iteration, as this would require manual selection of an appropriate resolution parameter interval. Instead, for each dataset, we used the average resolution parameter value from the initial consensus analysis. Each bootstrapped clustering result was then compared to the observed clusters using the Adjusted Rand Index (ARI) (package mclust, version 6.1.1). ARI quantifies the similarity between two clusterings while correcting for chance agreement. It ranges from −1 to 1, with 1 indicating identical cluster assignments and 0 expected by random labeling [33]. To ensure comparability, the observed network was subsetted to include only the nodes that remained after the attack. A high ARI score indicates strong similarity between clustering outcomes and, therefore, high resilience of network clusters.

**Figure 2:**
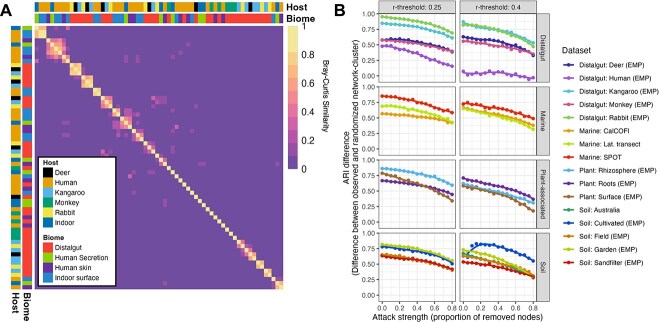
Stability of network clusters against node removal and compositional stability of clusters. (A) Compositional stability of microbial cohorts. Heatmap showing Bray–Curtis similarity of all cohorts identified within selected datasets (all datasets within the biomes distalgut, human skin, human secretion, and indoor surface). Most similarities between cohorts from unrelated biomes are low, indicating cohort specificity of ASVs. Analyses were conducted with r-threshold of 0.4. (B) Lineplot showing effect of increased attack-strength on ARI difference for selected datasets and for two different r-thresholds (see Fig. S4 for all datasets). Colours decode the different datasets.

To assess whether cluster resilience was statistically significant, we compared it to the resilience of a rewired network with identical structural properties. This was achieved by applying the same bootstrapped perturbation approach to 50 instances of randomly rewired networks that preserved node-degree distributions (10 x number of nodes rewire iterations). The ARI scores of the rewired networks were compared to those of the observed network by calculating their differences. A positive difference in ARI scores indicates that network clusters exhibit greater resilience compared to randomly rewired networks (Fig. 2A and B).

### Biogeographic properties of microbial cohorts

**Figure 3:**
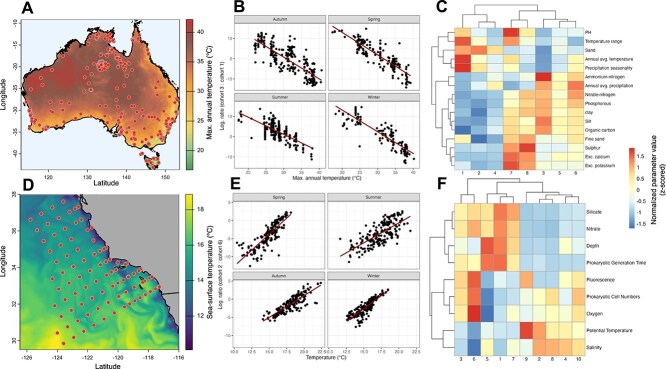
Biogeographical significance of microbial cohorts in soil and ocean environments. (A, D) Maps of sample-locations of two selected datasets: the Australian microbiome project data of prokaryotic soil communities (A) and CalCOFI data of free-living prokaryotic communities in a coastal ocean (D). The Australian map displays maximum annual temperature values obtained from the WorldClim database [38], while the marine dataset map illustrates the Californian coastline along with sea-surface temperature data retrieved from the Global Ocean Physics Analysis Forecast model (timepoint: 23.06.2023) via the Copernicus marine data storage web portal (data.marine.copernicus.eu). (B, E) Scatterplots of the composition of selected cohorts against environmental conditions for all samples in the respective dataset. Microbial cohorts in B and C were inferred from the same network, whereas cohorts in E and F were taken from Milke et al. [8] and represent open-ocean cohorts that were already described and validated. (C, F) Heatmaps representing environmental preferences for each microbial cohort based on measured environmental variables. All values were transformed into z-scores.

To investigate the biogeography of microbial cohorts, we selected two datasets with sufficient spatial coverage to enable a robust analysis of their spatial dynamics: the Soil, Australian Microbiome Project [21] and Marine, CalCOFI [22] datasets (Fig. 3A, D, visualized using the terra package in R (version 1.7–78)). To assess correlations between environmental conditions and cohort composition, we quantified microbial cohort abundances in the respective datasets. For the Australian soil dataset, we used network clusters derived from our clustering pipeline, whereas for the marine dataset, we used previously identified open-ocean cohorts from Milke et al. [8]. These ocean cohorts have been extensively studied regarding their biogeographic distribution and the ecological factors governing their spatial patterns. Cohort memberships were mapped to individual ASV sequences, and cohort abundances were determined as the sum of all member ASV abundances per sample, yielding sample-specific abundance values. Subsequently, for each sample, we computed abundance based log-ratios of selected cohorts (Australian Soil: cohort 3 and cohort 1; CalCOFI Marine: cohort 2 and cohort 6) and correlated these values with environmental variables (maximum annual temperature for the soil dataset and sea-surface temperature for the marine dataset; Fig. 3B, E). By using log-ratios of cohort abundances, we avoided compositionality biases when comparing between samples with different sequencing depths [34].

To characterize microbial cohort environmental preferences, we analysed associated environmental datasets. For the Australian soil dataset, we selected a subset of variables relevant to soil environments, supplemented with additional climatic variables obtained from WorldClim for the respective sample locations (specifically: annual mean temperature, precipitation seasonality, annual mean precipitation, and temperature range). For the ocean cohorts, we used environmental variables from the open-ocean dataset in which these cohorts were originally identified [8]. The predictability of microbial cohort composition based on environmental variables was based on permutational multivariate analysis of variance as implemented by the *adonis2* function in the vegan package. For that, we used a Bray–Curtis distance matrix for community data and a Euclidean distance matrix for the environmental data.

Environmental preferences of microbial cohorts were assessed by computing weighted means of each environmental variable, where weights were determined by cohort abundances in each sample. As indicated above, cohort abundances are determined as the sum of all ASV abundances associated to the respective cohort, yielding sample-specific abundance values. To facilitate comparison across variables, all values were transformed into z-scores. We visualized environmental preferences using heatmaps (Fig. 3C, F).

### Computing sample-wise modularity values

To assess the influence of environmental gradients on network structure, we calculated modularity for each sample individually. For this, we extracted a subnetwork containing only the ASVs present in the sample and computed its modularity using the cohort (cluster) assignments inferred from the full network.

### Verification against sample size and co-occurrence inference algorithm

We validated our cohort identification pipeline using subsets of the same datasets and three different co-occurrence inference algorithms. Our analysis focused on the Australian soil dataset and the open ocean dataset. The Australian soil dataset was subsampled three times, each using a subset of the total samples. Subsets corresponding to 50% and 70% of the full sample size were selected, with 50% subset representing a scenario of very sparse sampling. For the ocean dataset, we applied a systematic subsampling approach by separating Atlantic and Pacific Ocean basin samples. Each sample subset was filtered to include only ASVs present in all other subsets.

Co-occurrence networks were inferred for all sample subsets and the complete datasets using three inference algorithms: SparCC, SpiecEasi [35] (version 1.99.0), and Pearson correlation based on clr-transformed count data. For each subset-algorithm-combination, we clustered the resulting networks using either the previously described network clustering pipeline for SparCC and Pearson, or a greedy optimization algorithm [30] for SpiecEasi. This different treatment was needed because the standard clustering pipeline requires edge weights (e.g. SparCC or Pearson r-values), which SpiecEasi does not compute.

For network inference with SpiecEasi, we used neighbourhood selection with λ = 0.1. For SparCC, we applied an r-threshold of 0.4, and for Pearson, a slightly higher threshold of 0.5 to increase edge significance and reduce the likelihood of spurious connections [36]. The Pearson r-threshold of 0.5 was identified as producing clusters with similar sizes as the SparCC approach with r-threshold of 0.4.

To compare the resulting co-occurrence clusters with their corresponding clusters from other validation trials, we grouped them based on similarity. Specifically, we calculated the average ASV composition of each cluster (from the complete dataset) and computed Bray–Curtis dissimilarities to construct a hierarchical clustering dendrogram using the “ward.D” method. The optimal number of higher-order cluster groups (“cluster-clusters”) was determined using the silhouette width metric.

We further assessed sample-wise similarities by computing Bray–Curtis similarity between the cluster compositions of the complete dataset with SparCC co-occurrence algorithm and the verification samples.

## Results

### Microbial cohorts are ubiquitous

Microbial cohorts are encompassing units of microbial communities. Across all datasets and thresholds, network modularity was significantly higher than expected under randomized null models (*P* < .001), confirming that modular network structure is a pervasive feature of microbial communities (see Supplementary Table S2). The cohort structure was consistently inclusive of the majority of taxa. Microbial cohort-associated ASVs represented 75% of all counts at r = 0.4 in most datasets. As expected, the number of ASVs that can be assigned to a cohort increases further with decreasing correlation thresholds (90% at r = 0.25) (Fig. 1B).

We further compared inferred microbial cohorts between datasets using a conservative correlation threshold (r = 0.4) (Fig. 1). Microbial cohorts are ubiquitous features across environments, but exhibit biome-specific differences in size and diversity: Soil and plant associated samples harboured the most diverse cohorts (avg. richness for soil: 145 ASV; plant associated: 254 ASV), reflecting their generally higher community richness, whereas cohorts from human skin and secretions were up to six times less diverse (avg. richness for skin: 40 ASV; secretions: 62 ASV) (Fig. 1A). Indoor surfaces had the highest number of cohorts (avg. cohort richness 8.79), while human secretions had the fewest (avg. cohort richness 4.64) (Fig. 1D). Evenness-adjusted effective number of cohorts were highest in soil (avg. ESN 3.07) and on skin (avg. ESN 2.69) and lowest in secretions (avg. ESN 1.91) (Fig. 1E). These patterns were qualitatively robust across correlation thresholds (r = 0.25 and r = 0.4).

A systematic sensitivity analysis across r-thresholds (0.1–0.7) showed that increasing thresholds reduced noise and produced more clearly delineated networks with higher modularity but smaller cohorts in most datasets (Fig. S3). Although some datasets displayed specific and non-monotonic network responses, modular structure persisted across thresholds, indicating that while quantitative properties vary with threshold choice, modular organization is a consistent feature of microbial communities across environments. These findings highlight that correlation thresholds have variable impact on network topology and that advanced threshold selection approaches (e.g. applying random matrix theory [37]) can improve network reconstruction. However, because this study compares network structures across multiple datasets, we applied a consistent thresholding framework to avoid threshold-dependent biases and ensure comparability. Network metrics are also affected by the extent of cluster overlap [27], underscoring the need for clustering methods robust to ambiguous network structures, as implemented here.

The number of ASVs per cohort identified in our analysis far exceeds the number of organisms typically studied in cultivation-based experiments that study microbial interactions. A literature review of studies from the past two decades (Fig. 1C, F; Supplementary Table S3) highlighted that, with few exceptions, most studies relied on monocultures or co-cultures, while very few studies used mixed cultures comprising more than three species and even fewer analysed cultures with more than 10 species (Fig. 1C). Interestingly, in studies where more species were grown together, the focus of the studied ecological concepts shifted from “Cultivability,” “Mutualism,” and “Cross-feeding” to more complex ecological concepts like “Community functions,” “Complex interdependencies,” and “Stability interaction.” This highlights the key limitation of cultures involving only one or two species: their inability to observe emergent properties that arise at higher levels of organization, such as those found in microbial cohorts. Our literature review suggests that, in contrast to mono- or pairwise co-cultures, dilution culturing enables the study of interactions at a complexity level sufficient to approximate cohort dynamics under laboratory conditions.

### Stability of cohort composition

Several indicators suggest a high stability of the composition of an inferred cohort: First, composition was relatively stable across environments and hosts. Similar cohorts were inferred in markedly dissimilar biomes, such as indoor surfaces, human skin, and nasal mucus, despite their different environmental conditions. This stability extends even across studies: For example, a saliva-derived cohort closely resembled microbial cohorts found on skin and indoor surfaces (mean Bray–Curtis similarity between cohort composition 0.64) [31], despite originating from an independent dataset [32]. In the gut, cohort composition was highly conserved within related herbivores (kangaroos, rabbits, and deer; mean Bray–Curtis similarity between cohort composition 0.66) but distinct from human and primate gut cohorts (Fig. 2A). The low similarity between cohorts from unrelated biomes indicates that ASVs are largely cohort-specific. Secondly, the same cohorts are present in the ocean in regions that are not directly connected (see [8] and Fig. 5), indicating that community composition is deterministic. Hence, microbial cohorts represent non-random associations, specialized to distinct environmental conditions and occurring even in different biomes. Further, it supports the “key-lock” exclusivity of ASVs, indicating that specific microbial populations preferentially associate within particular cohorts, likely due to specialized metabolic dependencies or niche compatibility that limit associations outside these cohorts.

Thirdly, the network structure in microbial cohorts is internally robust against species loss. A systematic network attack (Fig. S2) revealed that cohorts persist despite node removal, suggesting strong internal connectivity (Fig. 2B & Fig. S4). Lower correlation thresholds (r = 0.25) yielded more stable clusters than higher thresholds (r = 0.4), likely due to generally larger cluster sizes. Across all datasets, all microbial cohorts were more robust than random expectations, reinforcing their biological relevance (Fig. 2B & Fig. S4).

### Distinct ecological preference of microbial cohorts

Microbial cohorts exhibit clear ecological preferences and biogeography, underscoring their ecological cohesiveness. Analysing their distribution in soil (Australian Microbiome Initiative) [21] and marine (CalCOFI) [22] datasets (Fig. 3A, D) revealed that cohort composition aligns with environmental factors. Marine cohort composition correlated with temperature, salinity, and nutrient levels (explaining 60% of total community variability; *P* < .001), while soil cohorts were structured by pH, climatic variables (selected from worldclim database [38]) and organic matter content (explaining 59% of total community variability; *P* < .001) (Fig. 3C, F). Interestingly, abundance-ratios between selected cohorts were linearly correlated with temperature conditions in both datasets (Fig. 3B, E). Abundance-ratios are especially relevant as they avoid compositionality bias when comparing between sequencing samples. These patterns highlight microbial cohorts as ecologically cohesive units whose composition is systematically shaped by environmental selection. Their deterministic behaviour in biogeographic studies suggests they could enhance species distribution modelling and improve our understanding of microbial dynamics across ecosystems.

**Figure 4:**
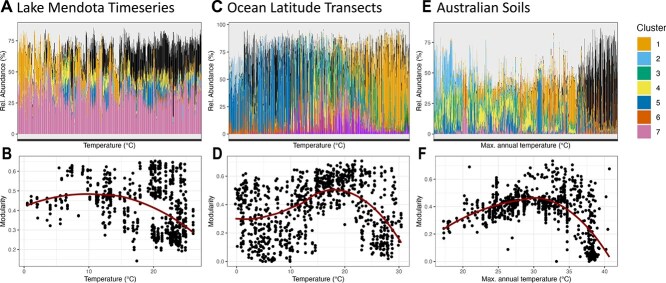
Composition of microbial cohorts along environmental gradients reflected by network modularity. (A, C, E) Barplot showing cohort composition of individual samples ordered by temperature conditions. Colours denote different cohorts, as indicated by the colour legend. Shown are three individual datasets for which environmental meta-data was available. (B, D, F) Scatterplots between temperature and network modularity. Each point depicts an individual sample and the modularity was calculated based on a subgraph including only network nodes of ASVs present in the respective sample. Red curves represent linear fits of 3rd degree polynomial formulas to the data points.

Building on this, we asked whether cohort dynamics could explain previously observed declines in modularity under stressful environmental conditions [39]. Across multiple datasets for which environmental metadata was available, microbial cohort composition shifted along gradients, with extreme conditions favouring fewer cohorts (Fig. 4A, C, E). In the Australian soil dataset, e.g. each of seven cohorts had a distinct temperature optimum. Recalculating network modularity in local subgraphs revealed a consistent decline in modularity at temperature extremes, coinciding with reduced cohort diversity (Fig. 4B, D, F). These findings suggest that modularity reflects the coexistence of multiple well adapted cohorts within a microbial community. As environmental stress intensifies, niche diversity declines, and a smaller set of tolerant cohorts dominates, resulting in lower network modularity. The reinterpretation of modularity as an emergent feature of microbial cohort overlap rather than a static feature of network structure [39] provides a new perspective on how environmental filtering shapes microbial networks. Since we observed these trends across datasets, our results reinforce the idea that modularity can serve as an ecological indicator of cohort or functional diversity [40].

### Robustness of inferred cohorts across sample sizes and network inference algorithms

**Figure 5:**
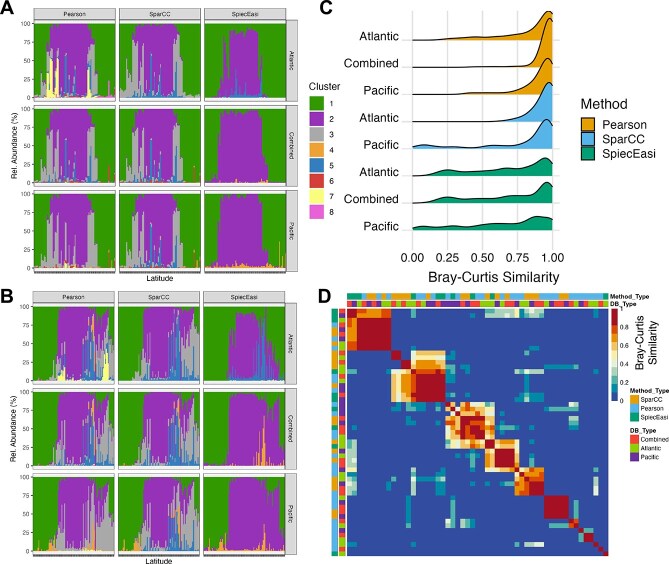
Verification of microbial cohort identification in the ocean. (A and B) Barplots of cohort composition across samples ordered by latitude in the Pacific (A) and Atlantic (B) oceans. Colours indicate cohorts; facets show co-occurrence algorithms (Pearson, SparCC, SpiecEasi) and data subsets (Atlantic, Pacific, combined). (C) Ridge plots of Bray–Curtis similarity between cohort compositions from verification runs and the gold standard (SparCC, combined dataset). (D) Heatmap of Bray–Curtis similarities between cohort compositions, with annotation colours marking cohort verification trials.

Overall, cohort identification proved robust across all algorithm-subset combinations, yielding highly comparable cluster compositions (Fig. 5, Supplementary Fig. S5 and S6). Cohort composition was more strongly influenced by the inference algorithm than by sample size, with SparCC and Pearson producing notably similar results (avg. ARI between “gold standard” and SparCC: 0.76; and Pearson: 0.81), while SpiecEasi deviated more strongly (avg. ARI of 0.45). Nevertheless, all three algorithms recovered the major cohorts, as reflected in their biogeographic distributions and Bray–Curtis similarities. SpiecEasi inferred the fewest cohorts, whereas Pearson identified the most (Fig. 5D).

Sample-wise comparisons of cohort composition showed high agreement between the “gold standard” (complete dataset using SparCC) and both SparCC and Pearson trials, with most Bray–Curtis similarity values exceeding 0.75. Agreement with SpiecEasi-based cohorts was weaker. Subsampling the Australian soil dataset to 50% of the total samples resulted in greater discrepancies between network inference algorithms, indicating that sparse sampling can obscure cluster boundaries and occasionally lead to merging or splitting of network clusters (Fig. S5 and S6). All algorithm-subset combinations revealed significant correlations between cohort composition and environmental gradients, with nearly identical linear slopes for SparCC and Pearson (Ocean ≈ 0.8; Soil ≈ −0.7) and moderately lower slopes for SpiecEasi (Ocean ≈ 0.5; Soil ≈ −0.3) (Fig. S7).

Taken together, these results confirm the robustness of our cohort identification pipeline, while highlighting that the choice of co-occurrence inference algorithm and sparsity of the dataset can substantially affect the number and quality of the identified cohorts.

## Discussion

### Co-occurring cohorts as ecologically meaningful units

Our analysis demonstrates that microbial co-occurrence networks consistently exhibit high modularity across diverse biomes, confirming that modularity is a general and robust feature of microbial community organization. This robustness was further supported by verification analyses across variations in sample size and network inference algorithms, which yielded consistent cohort structures and environmental associations. Conclusively, this modularity reflects the presence of stable, ecologically cohesive subcommunities, groups of microbial populations that not only co-occur more frequently than expected by chance but also persist across datasets and environments. These cohorts display biome-specific properties, with differences in size, richness and evenness shaped by environmental context, such as those observed between soil, marine and host-associated habitats. However, across all tested datasets, cohort composition correlates strongly with environmental gradients and remains conserved across independent datasets, suggesting that these assemblages are not merely statistical constructs but represent biologically meaningful and environmentally structured units.

In our analysis we only considered positive co-occurrences for cohort identification. While associations derived from co-occurrence analyses can include indirect links driven by shared environmental preferences, it is likely that organisms within the same co-occurrence cluster, i.e. sharing the same spatial and temporal distribution, interact in some way. These potential interactions are driven through widespread auxotrophies among microorganisms [7] and metabolic complementarity among co-occurring microorganisms [15, 16]. Consequently, many microorganisms depend on metabolic exchange with co-occurring populations to survive under specific environmental conditions [41, 42]. This constrain prevents isolation of many microorganisms, ultimately limiting classical cultivation approaches to a small subset of the total microbial diversity. However, overcoming this limitation is essential for enabling experimental validation of ecological implications of interactions within and between microbial cohorts. Hence, developing experimental approaches to sustain naturally occurring diversity will allow us to unravel the underlying interaction complexity and shed light on the evolutionary mechanisms that promote them. One such mechanism is division of labour, evolutionary promoted via genome streamlining or discarding distinct biosynthetic capabilities, that allows individual populations to optimize fitness through complementary functions within a group [43]. Consistent with this, widely distributed cohorts in freshwater systems were found to be composed mainly of microorganisms with smaller genome sizes and lower anabolic potential to synthesise amino acids and vitamins compared to less prevalent microorganisms [44]. Hence, it is reasonable to assume that positive co-occurrences imply potential functional relationships among cohort members. Further, the ubiquity and stability of microbial cohorts reflect the evolutionary trend toward genomic interdependencies among microorganisms, facilitating the evolution of auxotroph microbes [45] through their modular assembly.

The stability of cohort composition, even across distinct environments, and their strong association with environmental conditions underscore the ecological relevance of co-occurring microbial cohorts. In our analysis, cohorts often dominated under specific environmental conditions, with their composition closely aligned to environmental gradients such as temperature, pH, and nutrient availability. As proposed, tightly coupled metabolic interdependencies among cohort members synchronize their evolutionary trajectories and foster cohort stability, contributing to the observed clustering in microbial niche space [40, 46]. This clustered structure implies that the ecological processes environmental selection and dispersal jointly shape the biogeography of cohort members [8], explaining the consistent link between environmental gradients and cohort composition. Collectively, our analyses expand the interpretation of co-occurrence patterns from descriptive statistics to recognizing microbial cohorts as stable, and functionally cohesive ecological units.

### Microbial cohorts as a distinct level of microbial organization

**Figure 6:**
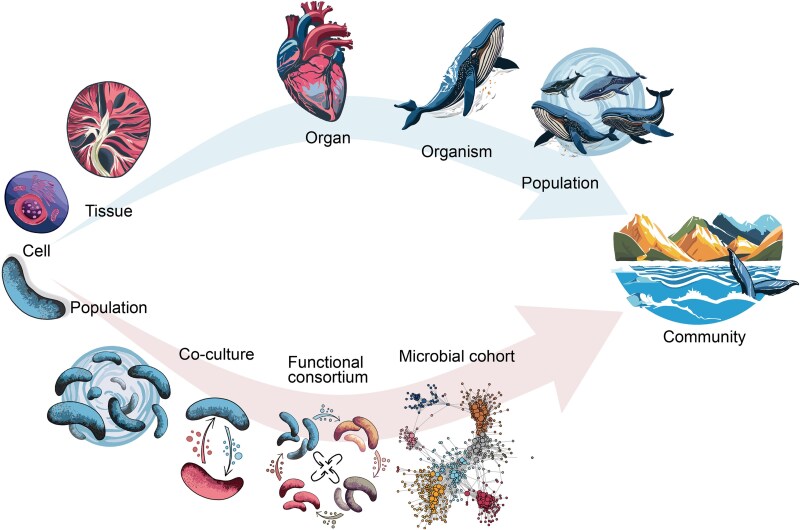
Concluding framework of (micro-) biological organization. Depicted is a linear hierarchy of co-evolutionary units.

Building on the inclusiveness, stability and coherent environmental preference, we propose microbial cohorts as a distinct level of microbiological organization (Fig. 6). The concept of hierarchical organization in biological sciences is a model of how living entities are organized and functioning [47]. Within this hierarchy, each level is formed by aggregating units from the level below, and novel properties can emerge at higher levels that are absent in the individual components. One emergent property that arises from biological organization is biological autonomy, typically associated with metazoan organisms. Biological autonomy is defined as the ability of an organism to maintain homeostasis internally while being able to interact with its environment externally [48]. Microbial cells exhibit a primordial level of autonomy as their cytosol is separated from the environment by the cell membrane. However, they are almost completely embedded in and interact with their environment including other microbial populations. Hence the autonomy of microbial populations is rather fuzzy and only interactions among various microbial populations enable microbial collectives to perform highly sophisticated tasks such as catalysing global biogeochemical cycles or breaking down food in the gut of mammals [49–51]. Given the deep embeddedness of microorganisms within their environment and the dependency on metabolic exchanges, we argue that cohort-level organization provides a meaningful scale at which emergent properties such as biological autonomy, collective resilience, and biogeochemical functionality manifest. Recognizing microbial cohorts as autonomous ecological entities may help reconcile the modular structure of microbial co-occurrence networks with their roles in ecosystem functioning, offering a lens through which to interpret microbial diversity beyond the level of individual taxa.

This perspective bears implications for practical approaches such as mixed-culture cultivation that better capture the dynamics and functional relevance of microbial cohorts. Thinking outside the classic approach and aiming to cultivate microorganisms in mixed cultures [52, 53] is crucial to start accounting for functional consortia and microbial cohorts, ensuring a more accurate representation of microbial interactions and their emergent potential. Insights gained from such cultivation techniques may be particularly relevant for community assembly studies, which have historically focused on the diversity of fewer organisms while often missing the broader diversity of microbial cohorts [51, 54, 55].

The interpretation of microbial cohorts as a distinct level of biological organization not only advances ecological theory but also has important implications for applied microbial ecology. Current predictive models such as species distribution models [56] or Earth system models [57], typically rely on either taxon-specific data or trait-based approaches focusing on resource competition, often overlooking the structured interdependencies among individual microbial populations. Our findings suggest that microbial cohorts, as stable and ubiquitous organizational units, represent a biologically meaningful and tractable level for modelling microbial responses to environmental change. Incorporating cohorts and associated metrics, such as modularity, could enhance the predictive power of these models by capturing emergent properties and ecological constraints that are not apparent at the level of individual taxa. Recently, such model approaches incorporating microbial community structure have become available [58], marking a shift toward more holistic representations of microbial community dynamics. In particular, the consistent association between cohort composition and environmental gradients implies that cohorts may serve as reliable ecological indicators within ecosystems. This cohort-based perspective also offers a potential bridge between taxonomic and trait-based frameworks once we assign functional roles to them. Integrating microbial cohorts into predictive modelling frameworks could improve our ability to forecast community dynamics, ecosystem functioning, and microbial responses to global change with greater accuracy and ecological relevance by implicitly considering biological interactions.

## Supplementary Material

ycag037_Supplemental_Files

## Data Availability

All data are derived from published sources. Accession numbers are referenced in the supplementary methods. Source-data for all figures are provided in Supplementary Table S4.

## Code availability

The code that was used in our analyses is provided at https://github.com/dermilke/Microbial_Cohorts and archived on Zenodo (https://doi.org/10.5281/zenodo.18310500).
