# Supplementary material for: Microbial cohorts: bringing ecological meaning to the modularity concept of co-occurrence networks
Source: ISME Commun. 2026 Feb 21;6(1):ycag037. doi: 10.1093/ismeco/ycag037 (PMC12981664; doi:10.1093/ismeco/ycag037)
Supplement: ycag037_Supplemental_Files [file ycag037_supplemental_files.zip › Supplementary_Material_ycag037.docx]

**Supplementary Material**

**Supplementary Figures:**

**Figure S1: Output of clustering pipeline. a** Metrics evaluating clustering performance across a range of clustering resolutions, illustrated with an example from the Marine: Comparison dataset. Penalized modularity, where higher values indicate better clustering performance. Silhouette width, with higher values representing an optimal balance between cluster cohesion and separation. Cluster member stability, where higher values reflect more consistent cluster assignments. Cluster size distribution, with lower values indicating more balanced cluster sizes. Optimal maxima or minima are marked by red points. In this example, we selected a clustering resolution range of 0.7 to 2.2 for the consensus analysis. **b** Consensus clustering networks, where each node represents an ASV and edges (grey lines) indicate >0 shared network clusters within the selected clustering resolution interval. Edge weights reflect the proportion of shared network clusters in the consensus analysis. Node colours represent network clusters identified using hierarchical clustering or network clustering. The bottom networks depict the observed co-occurrence networks, with nodes coloured according to the consensus clustering results. This example, based on the Marine: Comparison dataset, demonstrates that the network clustering approach provides a more coherent clustering of the consensus network.


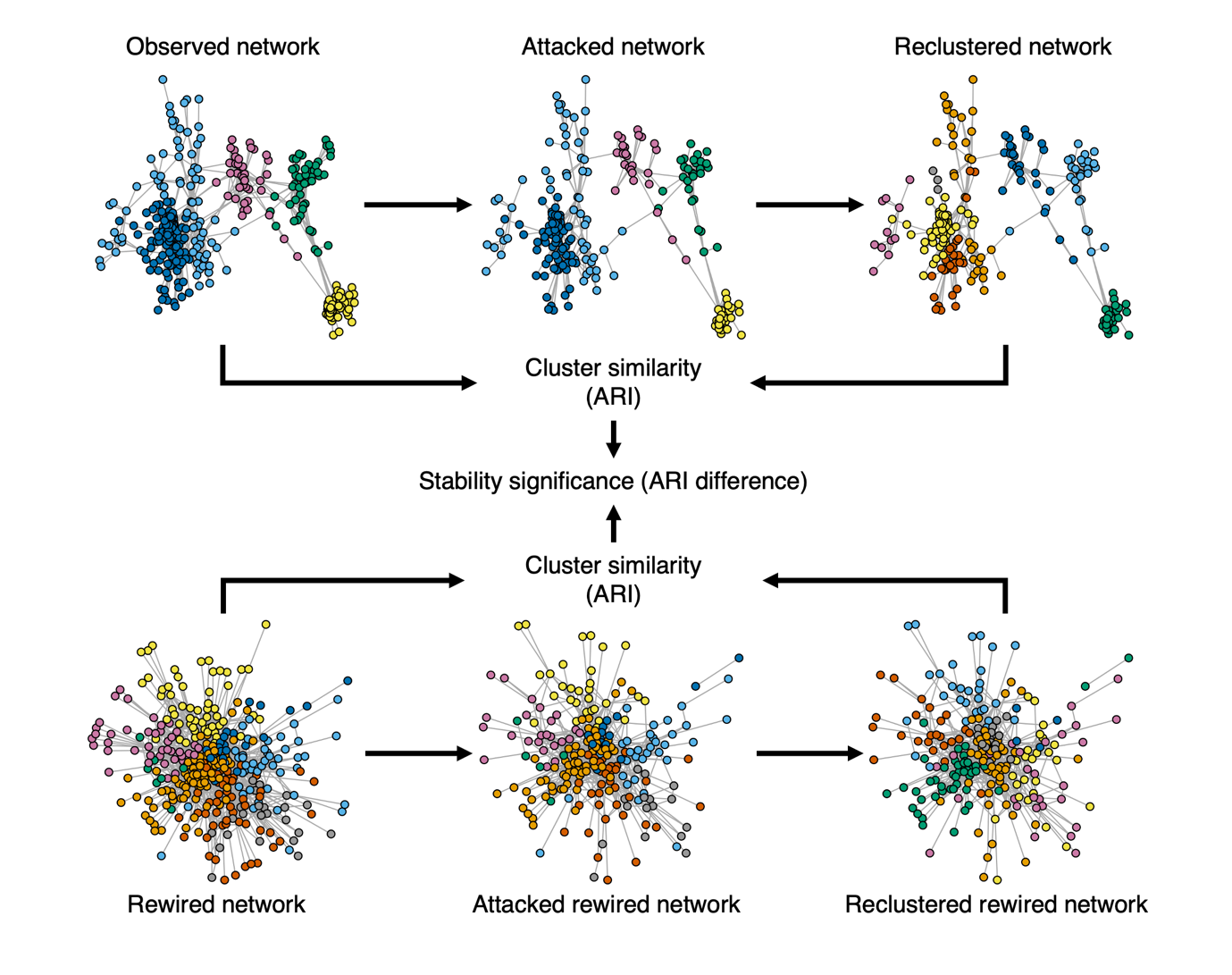


**Figure S2: Assessing cluster stability against network attack.** Depiction of the analysis framework to assess co-occurrence network stability. Node colours represent network-cluster membership. Top three networks show topology of an observed network that is first attacked and then reclustered based on the attacked topology. Bottom three networks show the same network with random rewiring (500 rewire-iterations) that is first attacked and then reclustered. For both, observed and rewired networks, similarity of cluster memberships between raw and attacked networks are computed based on Adjusted Ran Index (ARI) and their difference is interpreted as significance of cluster stability.

**Figure S3: Sensitivity analysis of varying r-thresholds on network properties.** The first column shows distribution of computed r-values in each dataset. The other columns show (from left to right) clustering coefficient, modularity, average number of members per cluster, and number of clusters within a network for varying r-thresholds. Each color depicts a different dataset (EMP = Earth Microbiome Project).

**Figure S4: Extended stability analysis of network clusters against node removal and compositional stability of clusters.** Lineplot showing effect of increased attack-strength on ARI difference for all datasets and for two different r-thresholds. Colours decode the different datasets. See Fig. S2 for a depiction of analysis workflow.

**Figure S5: Biogeographic verification of microbial cohort identification in Australian soil communities.** Barplots of cohort composition across samples ordered by max. annual temperature. Colours indicate cohorts; facets show co-occurrence algorithms (Pearson, SparCC, SpiecEasi) and random data subsets (keeping only 70% of samples). Left side shows results using 70% and on the right side 50% sample-subsets for network inference.

**Figure S6: Comparison of microbial cohort composition between verification runs.** Ridge plots of Bray-Curtis similarity between cohort compositions from verification runs and the gold standard (SparCC, complete dataset). Bray-Curtis similarity is computed sample-wise, by comparing the cohort composition of each sample from the verification runs with the corresponding sample from the gold standard run. Left panel shows cohorts inferred from co-occurrence networks constructed using 70% subsamples of the data, while right panel shows results based on 50% subsamples.

**Figure S7: Environmental responsiveness of cohorts from verification trials. a, b** Scatterplots showing log-transformed abundance ratios between two dominant cohorts in the (**a**) Ocean and (**b**) Australian soil verification datasets. In each case, the abundance ratio is plotted against the corresponding environmental variable: water temperature for (**a**) and maximum annual temperature at the sampling location for (**b**). Red lines indicate significant linear correlations between environmental conditions and cohort abundance ratios. While data subsets from verification trials did not significantly affect the slope or intercept of the linear models, the choice of co-occurrence algorithm influenced both parameters, with notable effects observed for SpiecEasi.

**Supplementary Methods**

**Collection and Curation of Amplicon Datasets**

To conduct robust co-occurrence network analyses, we compiled a diverse set of large-scale amplicon sequencing datasets that describe prokaryotic communities within specific environments. Given that co-occurrence inference requires a high number of samples to ensure statistical reliability, we selected datasets that contained a sufficiently large and coherent sample set from a given environment.

A primary resource for environmental microbiome data was the Earth Microbiome Project (EMP), which provides thousands of samples across various habitats ^1^. However, EMP datasets often integrate data from multiple studies, leading to heterogeneity that may confound co-occurrence analyses. For instance, the soil dataset within EMP includes samples from highly distinct environments, such as New York City gardens and sand filters for water purification. To mitigate this issue, we carefully filtered and subsetted the datasets to retain only those samples where co-occurences could establish.

In addition to EMP, we incorporated data from other large-scale sequencing projects of oceanic, soil and freshwater environments. Specifically, we selected datasets from the San Pedro Ocean Time Series (SPOT) ^2^, the Australian Microbiome Project ^3^, the Californian Cooperative Ocean Fisheries Investigations (CalCOFI) ^4^, comparable latitudinal ocean transects (short: comparison dataset) ^5^ and the Lake Mendota time series ^6^. These datasets offer temporal and spatial resolution of microbial organization in natural environments.

Collection and processing of datasets

Soil: Australia

We obtained a processed amplicon subset from the Australian Microbiome Project (version 2.3.0) via Bioplatforms Australia. The dataset was filtered using the following criteria:

- Environmental Broad Scale: "1 Conservation and Natural Environment"

- Depth: 0 to 0.1m

- Amplicon Target: 27f519r_Bacteria

Marine: SPOT (San Pedro Ocean Time Series)

- Data retrieved from ENA under accession numbers PRJEB48162 & PRJEB35673.

- Processed using the bioinformatics pipeline described in McNichol et al. ^7^ and Yeh et al. ^8^, following Milke et al. ^5^.

Marine: CalCOFI (California Cooperative Oceanic Fisheries Investigations)

- Accessed via ENA (PRJNA555783, PRJNA665326, PRJNA804265).

- Processed identically to SPOT data.

Marine: Comparison Dataset

- Includes latitudinal transects across the central Pacific and Atlantic Ocean basins.

- Further details available in Milke et al. (2022a; 2022b).

Freshwater: Lake Mendota Time Series

- Retrieved from ENA (PRJNA846788).

- Processed using the same pipeline as the SPOT, CalCOFI, and Marine Comparison datasets.

Earth Microbiome Project (EMP) Datasets

EMP datasets were accessed from the processed EMP dataset (release 1) via the FTP server (<ftp://ftp.microbio.me/emp/release1>; Thompson et al., 2017). The following subsets were selected:

Distal: Deer

- EMPO level 3: "Animal distal gut" & host: "sambar deer"

Distal: Human

- EMPO level 3: "Animal distal gut" & host: "human"

Distal: Kangaroo

- EMPO level 3: "Animal distal gut" & host: "kangaroo"

Distal: Monkey

- EMPO level 3: "Animal distal gut" & host: "spider monkey"

Distal: Rabbit

- EMPO level 3: "Animal distal gut" & host: "rabbit"

Freshwater: Germany

- EMPO level 3: "Water (non-saline)" & study ID: 945

Freshwater: Time Series

- EMPO level 3: "Water (non-saline)" & study ID: 1288

Freshwater: USA

- EMPO level 3: "Water (non-saline)" & study ID: 1883 & country: "United States of America"

Secretion: Saliva

- EMPO level 3: "Animal secretion" & description: "saliva" & host: "human"

Secretion: Nose:
- EMPO level 3: "Animal secretion" & description: "nose" & host: "human"

Skin: Foot:

- EMPO level 3: "Animal surface" & description: "foot" & host: "human"

Skin: Hand:

- EMPO level 3: "Animal surface" & description: "hand" & host: "human"

Indoor Surfaces

- EMPO level 3: "Surface (non-saline)" & study ID: 2192

Plant: Rhizosphere:

- EMPO level 3: "Plant rhizosphere" & description: "rhizosphere"

Plant: Roots

- EMPO level 3: "Plant rhizosphere" & description: "roots"

Plant: Surface

- EMPO level 3: "Plant surface"

Soil: Cultivated

- EMPO level 3: "Soil (non-saline)" & study ID: 1721 & collection timestamp: "16.11.11"

Soil: Field

- EMPO level 3: "Soil (non-saline)" & study ID: 990

Soil: Garden

- EMPO level 3: "Soil (non-saline)" & study ID: 1674

Soil: Sand filter

- EMPO level 3: "Soil (non-saline)" & description: "samples of sand from slow sand filter water purification system" & depth: "<= 0.4"

**Consensus Clustering Pipeline**

We developed a robust, semi-automated network clustering pipeline that identifies the most consistent clustering solution across a range of resolutions. The pipeline iterates clustering over a range of resolution values (0.1 to 5.0, in increments of 0.1) and evaluates clustering performance using the following metrics:

- Penalized modularity: Standard modularity adjusted for the standard deviation of cluster sizes, where higher values indicate optimal clustering.
- Silhouette width: Measures intra-cluster cohesion and inter-cluster separation; higher values indicate well-separated clusters.
- Adjusted Rand Index (ARI) between consecutive resolutions: Assesses stability by comparing cluster memberships across adjacent resolutions. Higher values indicate more consistent clustering.
- Cluster size distribution: Assessed via the coefficient of variation of cluster membership numbers. Lower values indicate more homogeneous cluster sizes, which aligns with our expectations.

These metrics were visualized in line plots across the resolution range, allowing users to identify an optimal resolution range for further consensus analysis (Fig. S1). The network was then re-clustered for each resolution value within this user-defined range, and cluster memberships for each resolution were stored in a table. Because cluster IDs are arbitrarily assigned, we could not easily count the number of cluster memberships for each ASV. Instead, we constructed a quadratic consensus matrix, where rows and columns represent network nodes. The matrix entries indicate the number of shared cluster memberships between nodes and are normalized by the number of clustering iterations.

This consensus matrix was subsequently used as an adjacency matrix to construct a weighted consensus network. The consensus network topology was visualized and clustered using short random walks (*cluster_walktrap* function). Given that clusters within consensus networks exhibited minimal overlap and were distinctly structured, the *walktrap* algorithm [30] was selected as the most effective clustering approach. Cluster memberships were visualized via node colours in both the consensus and original co-occurrence networks.

In rare cases, small spurious clusters emerged from the consensus analysis. To mitigate this, we evaluate the network clustering approach against a second approach with a user-defined expected number of clusters. This second approach uses hierarchical clustering to identify clusters within the consensus matrix. First, the consensus matrix was transformed into a distance metric, and hierarchical clustering was performed using the *ward.D2* method. The resulting dendrogram was cut at the height corresponding to the user-defined expected number of clusters. The hierarchical clustering results were visualized alongside network-based clustering for comparison (Fig. S1). We manually selected either the hierarchical or network clustering results as final consensus clusters, which most often aligned with the network-based approach. The final network clusters were exported for use in downstream analyses. A network cluster is in the following defined as a microbial cohort.

**Supplementary References**

1. Thompson, L. R. *et al.* A communal catalogue reveals Earth’s multiscale microbial diversity. *Nature* **551**, 457–463 (2017).

2. Yeh, Y.-C. & Fuhrman, J. A. Contrasting diversity patterns of prokaryotes and protists over time and depth at the San-Pedro Ocean Time series. *ISME Commun.* **2**, 36 (2022).

3. Bissett, A. *et al.* Introducing BASE: the Biomes of Australian Soil Environments soil microbial diversity database. *GigaScience* **5**, 21 (2016).

4. James, C. C. *et al.* Influence of nutrient supply on plankton microbiome biodiversity and distribution in a coastal upwelling region. *Nat. Commun.* **13**, 2448 (2022).

5. Milke, F., Meyerjürgens, J. & Simon, M. Ecological mechanisms and current systems shape the modular structure of the global oceans’ prokaryotic seascape. *Nat. Commun.* **14**, 6141 (2023).

6. Rohwer, R. R., Hale, R. J., Vander Zanden, M. J., Miller, T. R. & McMahon, K. D. Species invasions shift microbial phenology in a two-decade freshwater time series. *Proc. Natl. Acad. Sci.* **120**, e2211796120 (2023).

7. McNichol, J., Berube, P. M., Biller, S. J. & Fuhrman, J. A. Evaluating and Improving Small Subunit rRNA PCR Primer Coverage for Bacteria, Archaea, and Eukaryotes Using Metagenomes from Global Ocean Surveys. *mSystems* **6**, e00565-21 (2021).

8. Yeh, Y. *et al.* Comprehensive single‐PCR 16S and 18S rRNA community analysis validated with mock communities, and estimation of sequencing bias against 18S. *Environ. Microbiol.* **23**, 3240–3250 (2021).

9. Milke, F. *et al.* Composition and Biogeography of Planktonic Pro- and Eukaryotic Communities in the Atlantic Ocean: Primer Choice Matters. *Front. Microbiol.* **13**, 895875 (2022).

10. Milke, F., Wagner-Doebler, I., Wienhausen, G. & Simon, M. Selection, drift and community interactions shape microbial biogeographic patterns in the Pacific Ocean. *ISME J.* **16**, 2653–2665 (2022).
